# Optimizing Women’s Health in a Title X Family Planning Program, Baltimore County, Maryland, 2001-2004

**Published:** 2011-10-15

**Authors:** Diana Cheng, Priti Patel

**Affiliations:** Maryland Department of Health and Mental Hygiene; Center for Maternal and Child Health, Maryland Department of Health and Mental Hygiene, Baltimore, Maryland, and Scientific Education and Professional Development Program Office, Centers for Disease Control and Prevention, Atlanta, Georgia

## Abstract

**Background:**

Although women usually obtain family planning services during their reproductive years, their need for comprehensive preventive services that promote wellness beyond reproductive health is often ignored.

**Community Context:**

The Maryland Department of Health and Mental Hygiene sought to improve the general health of women and reduce their risk for adverse pregnancy outcomes by integrating women's health services into the Baltimore County Title X program. Title X is a federal family planning grant program primarily serving low-income, uninsured people.

**Methods:**

After completing a needs assessment, we addressed gaps in women's wellness services in 3 family planning clinics. On-site services included counseling, screening, and referral for nutrition and physical activity, adult vaccination, depression, domestic violence, smoking cessation, substance abuse, and general medical disorders. A local multidisciplinary task force provided leadership for the clinical infrastructure of the project and served as a resource for women's health referrals.

**Outcome:**

Every staff person surveyed reported that the project had a positive effect on the community and should be continued. Clients identified non–reproductive health services they needed but would not have received otherwise. During the 3-year period, patient volume increased 28% for the pilot sites, compared to 1% for the state family planning program overall.

**Interpretation:**

With collaboration from a multidisciplinary community task force, the Title X family planning program can help provide needed preconception, interconception, and general women's health services, especially for women who have difficulty accessing care.

## Background

Starting in early adolescence, girls and young women begin to seek reproductive health service practitioners (1) and rely less on their pediatrician or other primary care provider for health visits. However, to optimize health, most women of reproductive age must see different providers at different locations for primary care, prenatal care, and contraceptive visits, which can be inconvenient and expensive (2), especially for women who are uninsured or have difficulty accessing health care.

Because women spend most of their reproductive years in need of contraception, they usually prioritize reproductive health visits above other health care visits. In 2008, a study reported that 74% of women aged 15 to 44 years had received sexual or reproductive health care service (most commonly a Papanicolaou test, pelvic examination, or contraceptive service) during the previous year (3). Non–reproductive health care visits among young women often are deferred. In 2007, approximately 3 of 5 sexually active women aged 16 to 25 years enrolled in commercial health plans or Medicaid were not screened for chlamydia (4), as recommended by the US Preventive Services Task Force (USPSTF) in 1989. Researchers reported screening rates as low as 2% in 1 US managed health plan (5). Similarly, only 37% of patients received the recommended care as defined by quality indicators for sexually transmitted infections or vaginitis, and only 11% of patients received the recommended care for alcohol dependence (6).

These undertreated, underrecognized conditions, as well as other disorders (eg, depression, cigarette smoking, intimate-partner violence, and human immunodeficiency virus [HIV]), are more prevalent among women of reproductive age than any other age group. Additionally, such chronic conditions as obesity, hypertension, and diabetes can be prevented if healthy behaviors are initiated earlier in life.

## Community Context

The Women Enjoying Life Longer (WELL) Project was piloted by the Maryland Department of Health and Mental Hygiene (DHMH) as a women's health integration project at the 3 Title X family planning clinics in eastern Baltimore County and was eventually expanded to western Baltimore County. Eastern Baltimore County was selected because of its economically depressed, diverse populations just outside of the Baltimore city line and also because of the stability of the clinical staff in these 3 clinics, most of whom had been employed by the Baltimore County Health Department for more than 15 years. In addition, the WELL project director was a DHMH clinician who regularly provided care at 1 of these sites. A Title X program was chosen for the study because, as a federal grant program dedicated to providing family planning to low-income people, its patient population included young women who had difficulty accessing care; most were uninsured and had annual incomes below the federal poverty level (FPL). At the Baltimore County sites (east and west) in 2004, a total of 46% (n = 3,566) of the family planning clients traditionally came from minority populations: 34% were non-Hispanic black and 9% were Hispanic. One-third of the family planning patients in Baltimore County were younger than 20 years, and 11% were at least 35 years (Table). Eighty-six percent of the patients served by the Baltimore County family planning clinics had annual incomes at or below FPL, and 80% had no health insurance coverage. Clinical services were provided by a team of health counselors, nurses, nursing assistants, a physician, nurse practitioners, and an office assistant.

The primary objective of WELL was to improve the general health of young women and, if they became pregnant later, to help them enter pregnancy in a healthier state. The primary objective was to expand the scope of the Title X Family Planning Program to include nonreproductive preventive health services for young women without compromising the delivery of core contraceptive services. The new services were to be initially piloted in 3 sites in eastern Baltimore County, followed by expansion to the other Title X sites in western Baltimore County. A related secondary objective was to motivate and train the entire staff to expand their already overwhelming family planning workload to include new women's health services to the patient population. Patient and staff satisfaction with the newly expanded services and assessment of general women's health knowledge were the main outcome measures tracked.

## Methods

### Funding source

The Center for Maternal and Child Health, DHMH, was awarded funding for this project by the Maternal and Child Health Bureau, Human Resources and Services Administration. The total award for implementation of WELL was $100,000 annually, beginning in July 2001, for a 3-year period. The funding supported a half-time health educator position and paid for a needs assessment, evaluation, medical and educational supplies, and laboratory tests.

### Planning phase: 2001 to 2003

**1) Resource guide.** The Department of Health Education at Towson University, a state-affiliated university in Baltimore County, partnered with DHMH to complete a health resource guide for the pilot area in eastern Baltimore County. Such services as medical consultation, dental care, vision screening, HIV testing, radiology, laboratory testing, substance abuse treatment, mental health counseling, prescriptions, and domestic violence shelters were identified and classified in terms of cost, hours, languages spoken, pregnancy considerations, ease of transportation to site, and appointment availability. This guide was designed for use by WELL staff to provide referrals to women who needed outside services.

**Box 1. Women Enjoying Life Longer (WELL) Project Task Force**

**Table:** 

**Member**	**Consultative area**
Baltimore Medical System (federally qualified health center)	Medicine, psychiatry
Private practice physicians, Baltimore County (n = 2)	Medicine, family practice
Family Crisis Center of Baltimore County	Intimate-partner violence
Johns Hopkins Bayview Hospital (local hospital):Center for Addictions in Pregnancy	Substance abuse
Franklin Square Hospital (local hospital):Family practice, case management	Medicine, psychiatryHealth coverage
Baltimore County Health Department:Nutrition, mental health, vaccination, infectious disease, substance abuse, nurse practitioner, administration	Nutrition, physical activity, vaccination, smoking cessation, sexually transmitted infections, substance abuse
Center for Maternal and Child Health, Maryland Department of Health and Mental Hygiene	Women's health
Patients (n = 4), representing various age groups, races/ethnicities	Patient perspective

**Box 2. Women's Health Services Available Through the Women Enjoying Life Longer (WELL) Project, 2004**

**Table:** 

**Counseling/management, all women** Nutrition Body mass index Healthy eating, including folic acid and calcium Weight management (referral to Weight Watchers if desired) Physical activity Contraception^a^
**General medical screening (laboratory tests) — all women as noted** General history and physical exam,^a^ blood pressure^a^ Hemoglobin fingerstick,^a^ urine dipstick,^a^ pregnancy testing^a^ Cervical cancer screening^a^ (Papanicolaou, human papillomavirus) Sexually transmitted infections^a^ (chlamydia, gonorrhea, syphilis, HIV) Genetic screen^a^ (hemoglobinopathies, sickle cell anemia, phenylketonuria) Lipid profile, ages ≥20 y Glucose, ages ≥45 y Thyroid screening hormone, ages ≥35 y
**Smoking cessation, all cigarette smokers** Counseling Treatment (nicotine patches, nicotine gum, bupropion)
**Screening/counseling — all women** Depression, general — on-site treatment option Postpartum depression — on-site treatment option Premenstrual dysphoric disorder — on-site treatment option Intimate partner violence Substance abuse (alcohol, illicit drugs)
**Vaccination — all women at risk** Rubella^a^ Tetanus Hepatitis B

a Service was already part of family planning program before WELL program.

**2) Needs assessment.** Towson University conducted in-depth interviews with women residing in the pilot area (n = 20) and local health care providers (n = 10) to identify existing resources and women's unmet health care needs. Women volunteered for the focus groups in response to flyers posted in the clinics and community sites. Providers from health organizations listed in the resource guide were contacted and volunteered to be interviewed. Nearly all of the women (n = 19) identified depression and substance abuse as key psychosocial problems, followed by domestic violence and sexual abuse. Providers echoed the paucity of domestic violence, mental health, and substance abuse services and added nutrition and dental care.

**3) WELL Task Force.** A local task force was established in 2001 to provide leadership and consultation to WELL. The task force included Baltimore County program offices that were to be involved with WELL and related or interested community organizations (Box 1). Four patients, recruited by the clinical staff, also provided input.

**4) Selection of new services.** Using resources from USPSTF, American College of Obstetricians and Gynecologists, and National Women's Health Information Center (7-9), the WELL task force selected a core set of routine general health screenings, laboratory tests, and vaccinations for implementation (Box 2). All clinical services identified as gaps in the community needs assessment, except dental care, were added to WELL for screening, counseling, or treatment. The task force facilitated referrals for services unavailable at WELL.

**5) Staff training.** An essential element of WELL was staff training in all the new program areas (Box 2) that were to be integrated into the family planning program. A series of education presentations and workshops, including use of the behavioral stages of change (10), were held for the staff.

### Project implementation and evaluation: 2002 to 2004

**1) Full-service implementation.** A core set of women's health services (eg, adult vaccination, general screening for medical conditions, including chronic disorders, depression, substance abuse, and intimate-partner violence), counseling about nutrition and physical activity, and smoking cessation, were integrated into the family planning routines of the counselor, clinician, and exit nurse (Figure, Box 2). By 2004, WELL expanded to the Title X sites in western Baltimore County. A part-time health educator facilitated the flow of the clinic and helped the counselor with the more time-consuming activities (eg, weight management, smoking cessation, vaccinations, and other health counseling). Weight Watchers (Weight Watchers International, Inc, New York, New York) agreed to provide free registration and discounts for weekly sessions. WELL subsidized the cost to women so that no fees were incurred for 1 month of sessions. All women had their height and weight checked by a nursing assistant before seeing the clinician. During their annual examinations, all women also routinely received  a screening questionnaire for intimate-partner violence that they self-administered or completed with a counselor.

**Figure.**
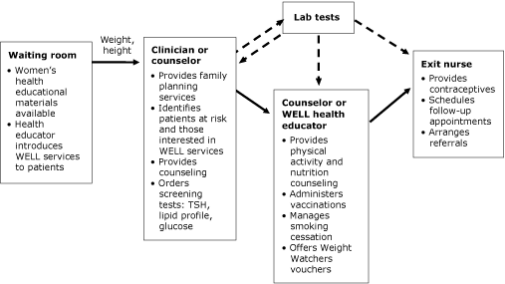
Women Enjoying Life Longer (WELL) service integration during a family planning visit. Abbreviation: TSH, thyroid-stimulating hormone.

Women were offered fasting lipid profile tests if they were at least 20 years old and fasting blood glucose tests if they were 45 years or older. Thyroid screening tests (thyroid stimulating hormone [TSH] levels) were routinely offered to women who were aged 35 years or older. All women, regardless of age, were offered these blood tests if they had any risk factors for the disorder (eg, obesity, family history, high blood pressure, or physical examination findings).

**2) Evaluation.** In 2004, the 11 family planning staff of eastern Baltimore County were given time during a staff meeting to complete a printed survey about the effect of WELL on the patients and staff. Also, 44 clinic users in eastern Baltimore County voluntarily completed printed surveys after their WELL clinic visit in June 2004. A 20-question quiz about women's health was attached to the user survey. This quiz served as a follow-up to a similar quiz given to 120 family planning clients who voluntarily agreed to be surveyed during December 2001, before WELL implementation. Certain questions were changed from the 2001 test because they were not clearly worded, and others were discarded from grading if the question or answer was open to interpretation. The surveys were analyzed by Towson University and DHMH.

## Outcome

**1) Increase in patient volume.** During the third year of WELL (2004), the 3 clinical sites provided services for 1,615 women, a 28% increase in patient volume from the 1,259 women served at those same sites in 2001 before WELL. No other changes accounted for this increase. During the same period, the patient volume increased 1% for the entire Maryland Title X Program.

**2) Laboratory test results.** A total of 351 laboratory blood tests were conducted by WELL clinics during 2004: 180 (51%) TSH, 110 (31%) fasting lipid profile, and 61 (17%) fasting glucose tests. A large percentage of these tests were abnormal, including 84% of fasting lipid profiles, 13% of fasting blood sugars, and 8% of TSH values. Data were not recorded on how many women were offered, refused, or agreed to the tests. Also, because of the need for 12 hours of fasting before the tests, most women did not return or forgot to fast for their tests.

**3) Body mass index and intimate-partner violence services.** A 2006 chart review of 306 women served at 1 of the clinic sites during a 6-month period in 2004 revealed that 30% were overweight (body mass index [BMI] 25.0-29.9 kg/m^2^), 29% were obese (BMI ≥30.0 kg/m^2^), and 21% had a history of physical abuse by a current or former partner. This chart review was completed for another research project and not for WELL.

**4) Evaluation: staff and patient survey of WELL.** In 2004, a print survey was conducted with the 11 family planning clinic staff involved with WELL. Responders were not identifiable. All staff believed that the program had a positive effect on the community and that WELL should continue. In addition, most reported that patients appreciated the additional services (n = 10), the clinic's ability to care for patients had improved because of WELL (n = 10), WELL did not interfere with family planning services (n = 9), and patients would be more likely to return to a clinic with WELL services (n = 8). In a discussion that followed, family planning staff voiced concerns that WELL services, however beneficial they were to the patients, were difficult to integrate without the help of an additional staff person. The WELL project coordinator, a health educator who was present at all WELL clinic sessions, was deemed essential to maintaining the flow of the clinic services.

Compared with 2001, more women in the 2004 WELL program correctly answered each of 15 questions that were identical on the 2 tests. For example, the percentage of women who knew that breast cancer was not the leading cause of death among women rose from 32% before WELL in 2001 to 80% after WELL. Women also increased their knowledge about the alcohol content of standard drinks (36% to 77%), osteoporosis prevention (19% to 68%), and daily calcium requirements (37% to 82%).

Survey questions in 2004 also included an assessment of patients (n = 44) about their satisfaction with the clinic site. When asked about their overall impression of the WELL program, most (n = 39) reported that WELL was excellent or very good, and the rest that it was good. Most women (n = 41) reported that the services they received were ones they would not have accessed if not for WELL.

### The WELL model in other parts of Maryland

One of the governor's strategic approaches initiated in 2008 to reduce infant mortality is focused on improving general health before pregnancy. Public health family planning clinics recently have begun to offer more comprehensive women's health services, similar to the WELL concept, in the 3 counties with the highest infant mortality rate.

## Interpretation

Since 1970, the Title X Family Planning Program has played a critical role in providing contraceptive and related preventive services to predominantly low-income and uninsured people (11). The WELL project's integration of comprehensive preventive women's health services into the family planning program allowed women who had difficulty accessing care a means of improving their health while taking care of their reproductive health needs. We determined that risk factors (eg, obesity, dyslipidemia, domestic violence) were already prevalent among young women at the WELL sites. Most women surveyed believed that WELL provided them with health care services that they otherwise would not have accessed. Their general knowledge of women's health also increased. Equally important, 8 of the 11 family planning staff surveyed believed that WELL did not interfere with delivery of family planning services. Furthermore, patient volume increased dramatically during the project period, perhaps reflecting the need for women's health services among this population as identified in the initial needs assessment. Women's health services also have the potential to expand reproductive health choices. For example, according to 1 WELL clinician's experience, smoking cessation allowed women aged 35 years or older to remain on oral contraceptives, and weight management improved continuation rates for women who experienced substantial weight gain with progestin-injectable contraceptives. Moreover, helping women leave a physically violent relationship liberated them from their partner's coercive demands on birth control use. Data from WELL did not capture the prevalence of these treatment results other than anecdotally.

The WELL project served as a bridge between family planning, prenatal, preconception, and interconception care, an ideal merger of the Title V, a federal block grant program to states for the improvement of maternal and child health, and Title X programs. Although optimizing perinatal health seems antithetical to a woman whose immediate objective is pregnancy prevention, the family planning visit may be the only opportunity that a woman has to receive advice about and treatment of conditions before pregnancy. The high unintended pregnancy rate in the United States of approximately 50% (12) indicates that integration of primary care, preconception health, and reproductive health at any time young women access health visits is beneficial. For women with difficult access to care, Title X programs and others that are publicly funded (eg, community or federally qualified health centers) can help integrate women's general and reproductive health (13,14). Adequate staffing and funding for these sites, perhaps through Medicaid expansion, may be necessary before any site can accommodate additional services.

In retrospect, we should have spent more time setting up a data system to capture the number of women who were eligible and participated in each of the WELL activities, along with their diagnoses, treatments, referrals, and follow-up. These data would have been useful for providing a cost-benefit analysis of the program, estimating the prevalence of acute and chronic disorders, and evaluating our ability to assess, treat, and refer women for non–reproductive disorder services in a family planning program.

In our experience with WELL, staff acceptance of the program was the most important ingredient for the project's success. During WELL's implementation, every staff member and administrative leader was enthusiastic about the women's health services and was an active participant in the decision-making process. As the natural turnover of staff occurred, the enthusiasm lessened. After the grant cycle was over, the budget for WELL was limited. Donated supplies (eg, nicotine patches, varenicline [a prescription drug approved for smoking cessation in 2006]) and funding for certain vaccinations have kept these services intact and strong. Family planning staff still offer nutrition education and health counseling; however, loss of the WELL health educator has made devoting the time desired for certain interventions challenging. Availability of other WELL services (eg, domestic violence or depression screening, treatment, and referral) remains entirely dependent on the individual health provider's interest and expertise. Routine population-based blood screening tests proved too costly to maintain.

WELL earned staff acceptance by training staff and encouraging their active participation in the decision-making process. Equally important, leadership from the multidisciplinary community task force facilitated for the staff the coordination of all new clinical services.

The integration of women's health with reproductive health services can provide young women a convenient way to optimize their general health, plan their pregnancies, and receive preconception and interconception services.

## Figures and Tables

**Table.** Demographic Characteristics of Title X Program Participants (n = 3,566), Baltimore County, Maryland, 2004

Characteristic	%
**Race/ethnicity**	
Non-Hispanic white	54
Non-Hispanic black	34
Asian	2
Hispanic	9
Other	1
**Age, y**	
20	33
20-34	56
≥35	11
